# Video assisted thoracoscopic resection of a posterior mediastinal Castleman's tumor

**DOI:** 10.1186/1749-8090-6-113

**Published:** 2011-09-20

**Authors:** Shohan Shetty, Robert A Brenes, Lucian Panait, Juan A Sanchez

**Affiliations:** 1 Saint Mary's Hospital, The Stanley J. Dudrick Department of Surgery, Waterbury, Connecticut, USA

## Abstract

Castleman's disease (CD) or angiofollicular lymph node hyperplasia is a rare spectrum of lymphoproliferative disorders. CD tumors are commonly localized in the mediastinum and are usually asymptomatic. The mainstay of treatment is surgical resection and has typically been performed using open thoracotomy. Few reports in the literature describe video assisted thoracoscopic resection of these tumors. The differential diagnosis for mediastinal masses is extensive, and CD tumors, although uncommon, should be considered. We describe a case report of a posterior mediastinal Castleman's tumor adherent to the esophagus, which was resected thoracoscopically and review the literature.

## Introduction

First described in 1954, Castleman's disease (CD)[1] is a rare lymphoproliferative disorder involving lymphocyte proliferation and excessive cytokine production. The characteristic lymphoid tumors may occur singly or in a multicentric pattern, the latter being more commonly associated with signs and symptoms including fever, weight loss, anemia, anorexia, and low white blood cell count.

Surgical resection is the mainstay of treatment for unicentric mediastinal CD[2] and has been typically performed via standard thoracotomy[3-5]. While video-assisted thoracoscopic surgical (VATS) resection has become an effective and reliable option for excising mediastinal masses[6,7], very few reports describe resection of mediastinal CD using this approach[8-11]. Because these tumors are highly vascular and often have dense adhesions to the surrounding tissue, they must be approached with great care especially in the mediastinum given the close proximity to vital structures[4,12]. We report our experience with a rare posterior mediastinal CD tumor adherent to the esophagus and review the literature.

## Case report

**Figure 1:**
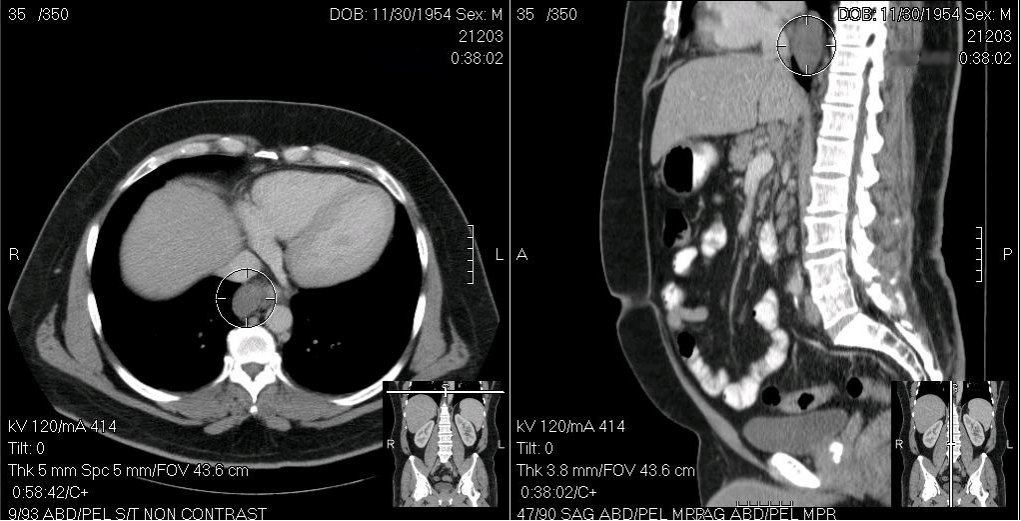
**CT scan images of the posterior mediastinal mass**.

A 54-year-old Hispanic male presented to the emergency department with abdominal pain. He denied any history of fever, night sweats, weight loss or fatigue. He denied any dysphagia but complained of mild anorexia. Physical examination was essentially normal except mild tenderness over the spleen. Complete blood count and comprehensive metabolic panel were within normal limits. A contrast-enhanced computed tomographic scan of the abdomen and pelvis incidentally detected a posterior mediastinal mass measuring 5 × 3 × 2 cm as well as moderate splenomegaly (Figure 1). There was no radiological evidence of invasion of the adjacent vasculature, heart, diaphragm or bony tissues. A positron emission tomography (PET) scan was not performed.

**Figure 2:**
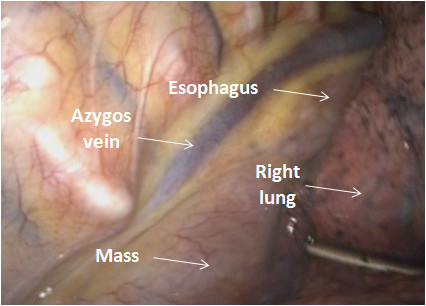
**Thoracoscopic view of posterior mediastinal structures**.

**Figure 3:**
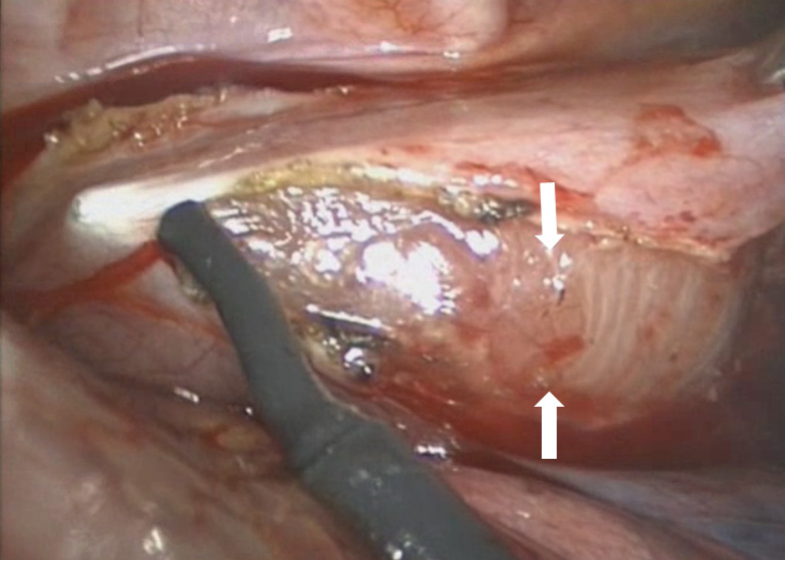
**Thoracoscopic view of the posterior mediastinal mass with the edges of the mediastinal pleura divided (arrows)**.

Complete surgical resection using VATS was undertaken under single-lung anesthesia using a 10-mm and two 5-mm ports. The mass was visualized and located between the azygous vein and the esophagus (Figure 2). The parietal pleura was incised and the 5 × 2.5 × 1.5 cm densely adherent and highly vascularized mass (Figure 3) was separated from the esophagus using electrocautery as well as sharp and blunt dissection. The thoracic duct located adjacent to the mass was ligated securely. An esophageal leak test using saline in the pleural cavity and air insufflated into the esophageal lumen confirmed the integrity of the esophageal mucosa. The patient's postoperative course was entirely uneventful. A serum analysis for HIV infection was negative. Pathologic examination of the dark brown, encapsulated mass confirmed the diagnosis of hyaline-vascular CD, an unexpected diagnosis. The patient has been asymptomatic on the subsequent follow-up. His initial abdominal pain did not recur. It is unclear whether the patient's splenomegaly was related to CD.

## Discussion

Castleman's disease (CD), also known as angiofollicular lymph node hyperplasia, giant lymph node hyperplasia, lymph node hamartoma, as well as benign lymph node lymphoma, was first described by Dr. Benjamin Castleman in a patient with solitary mediastinal lymph nodes in 1954[1] and in a group of largely asymptomatic patients with benign mediastinal lymphadenopathy in 1956[13]. It is now considered part of the uncommon spectrum of lymphoproliferative disorders which have several distinct pathological and clinical variants[14].

Previously classified broadly into unicentric and multicentric types, recent authors favor defining CD by histopathogenic type into hyaline-vascular CD, plasma cell CD, human herpes virus-8 (HHV-8) associated CD (also known as plasmablastic CD) and multicentric CD, not otherwise specified. The hyaline-vascular CD accounts for approximately 70% of patients with CD and involve men and women equally[15].

While the etiology is uncertain, pathogenic factors such as HHV-8 and interleukin-6 (IL-6) have been associated with CD. The majority of HHV-8 associated CD is multicentric and has a risk of progression to large B-cell lymphoma. Human immunodeficiency virus (HIV) positive patients with CD are almost always coinfected with HHV-8. Although the role of IL-6 in the pathogenesis of CD is unclear, the systemic symptoms associated with CD have been linked to overproduction of IL-6. In addition, viral IL-6 has known hematopoetic and angiogenic effects[14] and treatment with anti-IL-6 antibodies have shown promising results[16].

Although the mediastinum is the most common location for unicentric CD, it has been noted to occur in the cervical, axillary and abdominal regions[2,13,14]. Systemic symptoms are unusual in the hyaline-vascular CD[14]. Mediastinal CD can cause esophageal varices resulting from abnormally large amount of drainage into esophageal veins. In addition, mediastinal masses have been known to cause displacement and compression of the esophagus resulting in dysphagia [17].

Multicentric CD can present as multifocal lymphadenopathy and hepatosplenomegaly is common[14]. In plasma cell CD and multicentric CD constitutional symptoms (fever, night sweats, fatigue and weight loss) and laboratory abnormalities (anemia of chronic disease, elevated erythrocyte sedimentation rate, thrombocytopenia, deranged liver function tests, hypoalbuminemia) can occur[14].

Radiologic evaluation mainly involves computed tomography (CT), magnetic resonance (MR) imaging and positron emission tomography (PET/CT). The typical feature on CT scan is a well circumscribed mass with soft tissue attenuation and rarely calcification. On MR imaging, these highly vascular tumors appear solid and have intermediate to high signal compared to muscle on T1 weighted images. Hyper intense signal is seen on T2 weighted images. PET scan is a useful modality for staging and monitoring response to chemotherapy[18].

This case appears to represent an example of unicentric disease even though the spleen was enlarged, particularly since the patient did not have the characteristic constitutional symptoms of multicentric disease. Treatment options for unicentric CD include surgical resection [1,2,13] and irradiation when surgery is not an option[14]. Radiotherapy has been associated with acute and late toxicities[19] as well as stenosis of the esophagus, trachea and the bronchus when used for mediastinal CD tumors[20]. Preoperative embolization can be attempted prior to surgical resection to minimize intraoperative bleeding from hypervascular mediastinal CD tumors[21]. Multicentric CD requires systemic therapy which can include chemotherapy, antiviral therapy, steroids and the use of a humanized monoclonal antibody to IL-6 receptor[14].

The differential diagnoses for posterior mediastinal masses are extensive and include neurogenic tumors, sarcomas, lymphomas, pleural based tumors, bronchogenic cysts, enteric cysts, teratomas, arteriovenous malformations and metastatic masses[8]. These tumors, although uncommon, should be included in the differential diagnoses and must be especially considered in the presence of constitutional symptoms associated with CD. Surgical resection of isolated posterior mediastinal masses, as in this case, is usually indicated to exclude malignancy and to prevent local mechanical effects.

Diagnostic modalities in addition to imaging, for posterior mediastinal masses include ultrasound guided endoscopic fine needle aspiration and percutaneous transthoracic core biopsy. However, these can be technically difficult and specimens obtained by these techniques may not yield sufficient material for an accurate diagnosis. Excisional biopsy, particularly in unicentric disease, is preferred in order to assess cell type and tissue architecture, but can be associated with substantial bleeding as these tumors are known to be highly vascular[8].

The technical aspects of this case underscore the characteristic features of this tumor and its location. For example, we elected to ligate the thoracic duct early and intentionally because its integrity could not be assured given its location and the extensive dissection required, thereby decreasing the risk of a postoperative chylothorax, a known complication of posterior mediastinal surgery[22]. Knowledge of thoracic duct anatomy, vigilance during dissection, prompt recognition of duct injury can dramatically reduce the incidence of a postoperative chylothorax. In addition, adherence of the tumor to the esophagus demanded that we exclude esophageal damage at completion of the resection. The large number of lymphatic channels coursing through the surface of the tumor requires ligation with multiple clips since electrocautery should not be solely relied upon to prevent lymphatic leakage.

## Conclusion

We describe resection of a highly vascular posterior mediastinal Castleman's tumor with dense adhesions to the esophagus employing a minimally-invasive approach. Our case confirms that, using meticulous dissection and hemostasis, VATS resection can be performed safely. Particular attention to the thoracic duct with a view towards early ligation is necessary to prevent postoperative chylothorax. When the tumor is adherent to the esophagus, an esophageal leak test should be performed upon completion to assess the integrity of the esophagus. Minimally invasive thoracoscopic resection is a safe and feasible option in the management of posterior mediastinal CD. Mediastinal CD, although uncommon should be included in the differential diagnoses of posterior mediastinal tumors.

## Consent

Written informed consent was obtained from the patient for publication of this case report and accompanying images. A copy of the written consent is available for review by the Editor-in-Chief of this journal.

## Competing interests

The authors declare that they have no competing interests.

## Authors' contributions

Case report conception and design: SS, RAB, LP, JAS. Acquisition of data: SS, RAB. Drafting of manuscript: SS, RAB, LP. Critical revision: JAS. All authors read and approved the final manuscript.
